# Exploring Agentic AI in Healthcare: A Study on Its Working Mechanism

**DOI:** 10.3389/fmed.2025.1753443

**Published:** 2026-01-28

**Authors:** Parvathaneni Naga Srinivasu, Gorli L. Aruna Kumari, Shakeel Ahmed, Abdulaziz Alhumam

**Affiliations:** 1 Amrita School of Computing, Amrita Vishwa Vidyapeetham, Amaravati, Andhra Pradesh, India; 2 Department of Computer Science and Engineering, Gitam School of Computer Science and Engineering Gitam Deemed to be University, Visakhapatnam, India; 3 School of Computer Science, Taylor's University, Subang Jaya, Selangor, Malaysia; 4 Department of Computer Science, College of Computer Sciences and Information Technology, King Faisal University, Al-Ahsa, Saudi Arabia

**Keywords:** 6G, Agentic AI, federated learning, healthcare, SWOT analysis

## Abstract

**Introduction:**

Rapid advancements in artificial intelligence (AI) have ushered in an era of hyperautomation and intelligent orchestration across multiple engineering domains, with healthcare emerging as one of the most impactful application areas. Among recent developments, Agentic AI has gained attention as a sub-domain of AI capable of autonomous operation, decision-making, and goal-driven behavior with minimal human intervention. This study aims to explore the architectural and functional role of Agentic AI in modern healthcare systems.

**Methods:**

The study adopts a conceptual and analytical approach to examine the core components of Agentic AI, including agent design, decision-making mechanisms, task allocation strategies, agent coordination, and ranking frameworks. It further investigates the integration of emerging 6G networking technologies within Agentic AI architectures. A qualitative case study on remote robotic surgery is presented to illustrate practical applicability. Additionally, a Strengths, Weaknesses, Opportunities, and Threats (SWOT) analysis is conducted to assess strategic and operational considerations.

**Results:**

The analysis demonstrates that Agentic AI architectures, when supported by high-speed and low-latency 6G communication, can enable efficient autonomous decision-making and coordinated task execution in complex healthcare workflows. The case study highlights the feasibility of Agentic AI in enabling remote robotic surgery with enhanced responsiveness, precision, and reliability. The SWOT analysis reveals strong potential for scalability and efficiency while also identifying challenges related to ethical governance, system robustness, and security.

**Discussion:**

The findings suggest that Agentic AI represents a promising paradigm for next-generation healthcare systems, particularly in remote and critical care applications. While the proposed framework offers architectural insights and strategic value, responsible integration requires addressing limitations such as trust, regulatory compliance, and system transparency. Overall, this study provides a holistic understanding of how Agentic AI can be effectively and ethically integrated into healthcare ecosystems.

## 1 Introduction

Healthcare systems across the world are becoming increasingly challenged with the growing patient volumes, limited clinical resources, and the need for timely and personalized care delivery. AI has emerged as a key enabler for addressing all such challenges by improving the efficiency and precision of clinical and administrative processes (1). However, conventional AI approaches are largely dependent on predefined inputs and static decision pathways, which restrict their effectiveness in dynamic healthcare environments where real-time adaptability is essential. This limitation has created the need for more advanced intelligent systems that are capable of autonomous reasoning and contextual decision-making (2).

**Figure 1:**
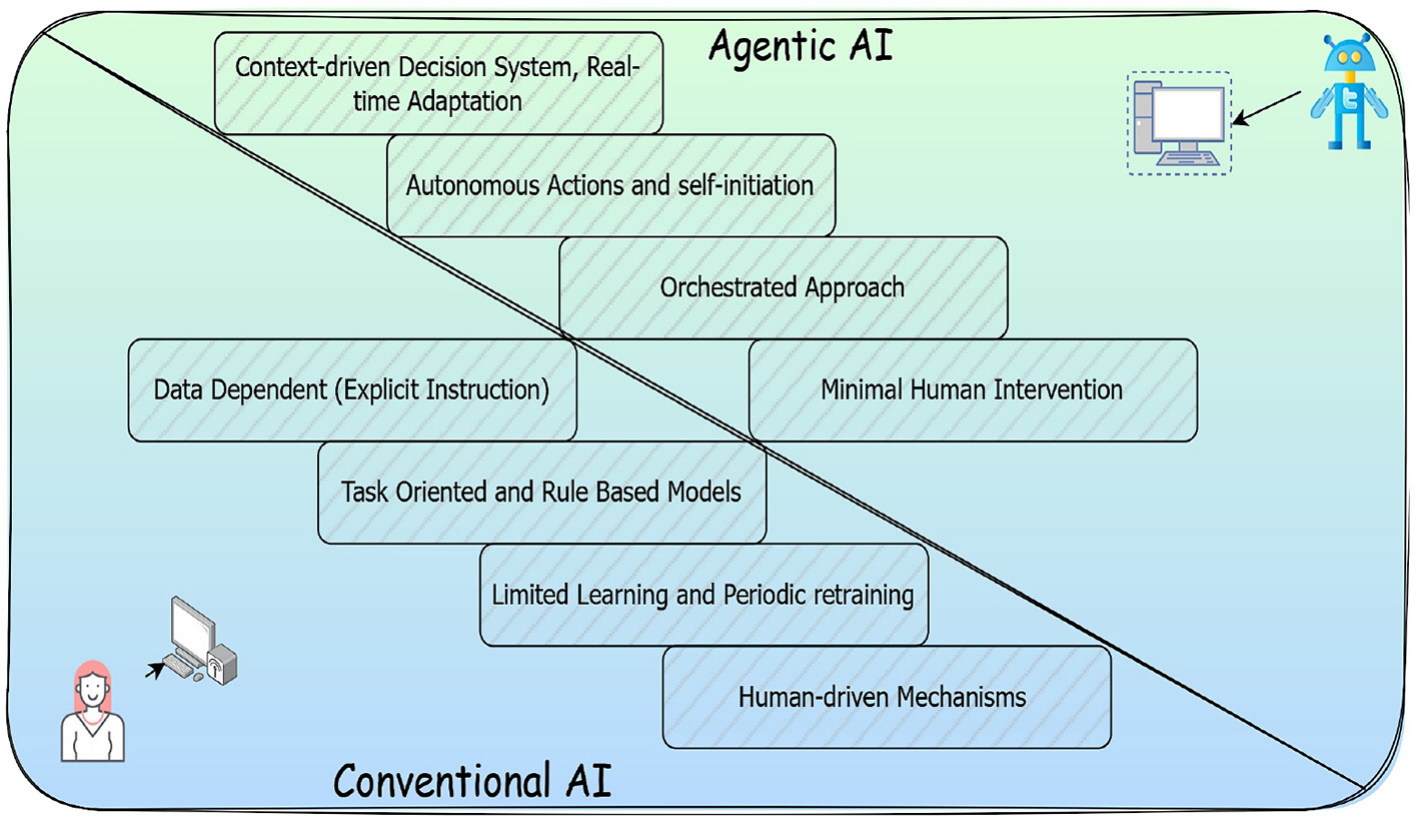
Features of conventional and Agentic AI applications. Created with Diagrams.net.

Agentic AI is the most recent advancement in this direction, offering autonomous task generation, complex problem-solving, and real-time decision-making capabilities (3). These systems can process heterogeneous clinical data, adapt to evolving situations, and refine their behavior through the process of continual learning. As a result, Agentic AI has the potential to enhance various healthcare functions all the way from clinical decision support to operational management by significantly reducing the human workload and improving care quality (4). The conceptual distinction between conventional AI and Agentic AI is illustrated in Figure 1.

Healthcare systems face increasing demands, resource limitations, and the need for more efficient, accurate, and personalized care delivery. It can impact the various sub-domains of the healthcare domain, ranging from automating complex enhanced clinical decision-making processes to administrative workflows such as scheduling and billing. For instance, the agentic systems could proactively monitor patient data streams from wearable devices and synchronize with electronic health records, thereby autonomously adjusting personalized treatment plans based on real-time responses and assist in identifying potential health crises before the issue escalates. Agentic AI also assists in handling tasks such as managing hospital resources and organizing patient information. This would reduce the workload of healthcare professionals and it could lead to faster, more accurate care for patients. Furthermore, Agentic AI could accelerate drug discovery and development by autonomously designing, executing, and interpreting experiments. The following are the contributions of the present study.

- The present examination highlights some of the fundamental differences between conventional AI and Agentic AI. It also highlights various phases of the Agentic AI.
- The examination outlines the generic architecture of Agentic AI and the working of the model.
- The examination has discussed the architecture of Agentic AI in the healthcare domain and has discussed how various components of the model would work collaboratively to perform a task.
- The case study on remote robotic surgery over the 6G networking technology using the Agentic AI is being presented and the roles of various agents are being discussed.
- Strengths, Weaknesses, Opportunities, and Threats associated with the Agentic AI in the healthcare domain are being highlighted.

The present discussion is primarily conceptual, proposing an architectural framework and outlining multi-agent workflows to illustrate the potential role of Agentic AI in healthcare. The contribution focuses on synthesizing existing knowledge and presenting a structured perspective that may guide future implementation and empirical investigation.

## 2 Background

The present section provides the trade-off between conventional AI and Agentic AI technologies.

### 2.1 Trade of between conventional models and Agentic AI

The trade-off between conventional AI and Agentic AI lies primarily in the balance between control, complexity, and autonomy. More human intervention is involved in the conventional AI, and the conventional models are data-driven models, which are largely used for data analytics and pattern analysis (5). However, the conventional AI models lack the flexibility to adapt beyond their programmed scope, and they are also suitable only for narrow tasks where rules and outcomes are clearly defined. On the other hand, Agentic AI models are autonomous and adaptable in nature, thereby allowing the systems to operate with minimal supervision, generate their own goals, and respond dynamically to changing environments. The flexible approach in Agentic AI would result in developing a realistic model that is cognitive.

The major trade-off lies in building the models. Obviously, the Agentic AI is more complex to build and implement. These models require substantial computational and infrastructural resources for development and deployment, which would incur a high operational cost. This shift in autonomy introduces a new layer of responsibility in ensuring ethical design, robustness, and interpretability of the model being built (6). The technological trade-off among the conventional and Agentic AI applications is shown in Table 1.

**Table 1:** Comparison between conventional AI and Agentic AI.

**References**	**Dimension**	**Conventional AI**	**Agentic AI**
Ren et al. (7)	Adaptability	Limited to predefined tasks and static rules	Adapts dynamically to evolving environments and goals
Nisa et al. (8)	Autonomy	Requires predefined inputs and task boundaries	Generates tasks, decisions, and strategies autonomously
Sai et al. (1)	Data dependency	Strong reliance on fixed training data and static models	Integrates real-time perception, reasoning, and planning
Alam and Khan (2)	Decision-making	Deterministic or model-bounded decision rules	Context-aware, multi-agent reasoning mechanisms
Hosseini and Seilani (9)	Resource requirements	Moderate computational complexity	High computational and orchestration demands
Karunanayake (10)	Healthcare applicability	Suitable for prediction and classification tasks	Effective for autonomous monitoring, workflow automation, adaptive planning

### 2.2 Phases and key components of Agentic AI

The operational workflow of Agentic AI can be conceptualized as four interrelated phases, which include the perception phase, reasoning and planning phase, action phase, and learning and feedback phase. Each of the phases has its significance in the entire life cycle of the Agentic AI model. The significance of each of the phase is listed below as a bullet point. The phases and the corresponding tasks of each phase are presented in Table 2.

**Table 2:** Operational phases of an Agentic AI system.

**References**	**Phase**	**Description**	**Technologies**
Sapkota et al. (11)	Perception	Acquires and processes multimodal data from sensors, signals, and digital inputs	NLP encoders, signal processing, multimodal feature extractors
Borghoff et al. (12)	Reasoning and planning	Interprets data, infers goals, and formulates action sequences	Knowledge graphs, rule-based systems, probabilistic reasoning
Brohi et al. (13)	Action execution	Executes planned operations and interacts with external digital or physical systems	API integrations, robotic control modules, workflow automation engines
Olujimi et al. (14)	Learning and Feedback	Refines internal models through continuous evaluation, feedback, and adaptation	Meta-learning, reinforcement learning, continual learning frameworks

- The perception phase involves data collection and interpretation from various sources in the environment. Depending on the application, this may include sensor inputs, digital logs, user interactions, video feeds, or unstructured text. The system employs data acquisition tools, signal processing, and data analytics to extract meaningful insights from the input data. The current phase is significant in building the context-sensitive representation of the current environment.
- Reasoning and planning phase evaluates the collected data, identifies objectives, and formulates future action plans. This is achieved through the use of knowledge representations such as the knowledge graphs, rules, and logic-based inference. The probabilistic reasoning and decision-making strategies are being used in formulating the action. These mechanisms are being used to leverage the most suitable action.
- Action phase would execute the plan, which includes several sub-tasks such as managing the workflows and interactions within the system and with the external entity. The corresponding outputs are generated when performing the corresponding action. Effective coordination and task execution are crucial in ensuring that the model fulfills its objectives efficiently.
- The final phase is the feedback collection phase, which involves evaluating the outcomes of the model's actions and incorporating feedback to improve future performance. It also collects the data on success metrics, system responses, and environmental changes. This is a continuous process, where the outcome would assist in making better-suited actions in the future to effectively handle complex problems.

### 2.3 Functioning of Agentic AI

Agentic AI systems heavily rely on these modern AI technologies such as Large Language Models (LLMs) (15), Generative AI (16), Generative Pre-trained Transformer (GPT) (16), and other advanced natural language processing techniques (17), especially as tasks become more complex and require interaction in human-centric domains. Agentic AI operates by integrating autonomous AI agents, and the operation of Agentic AI entails a set of structured interactions and coordinated processes. Autonomous agents continuously perform environmental sensing, acquiring data from both digital infrastructures and physical systems. This real-time data acquisition is fundamental to maintaining an accurate and dynamically updated model of the operational context.

**Figure 2:**
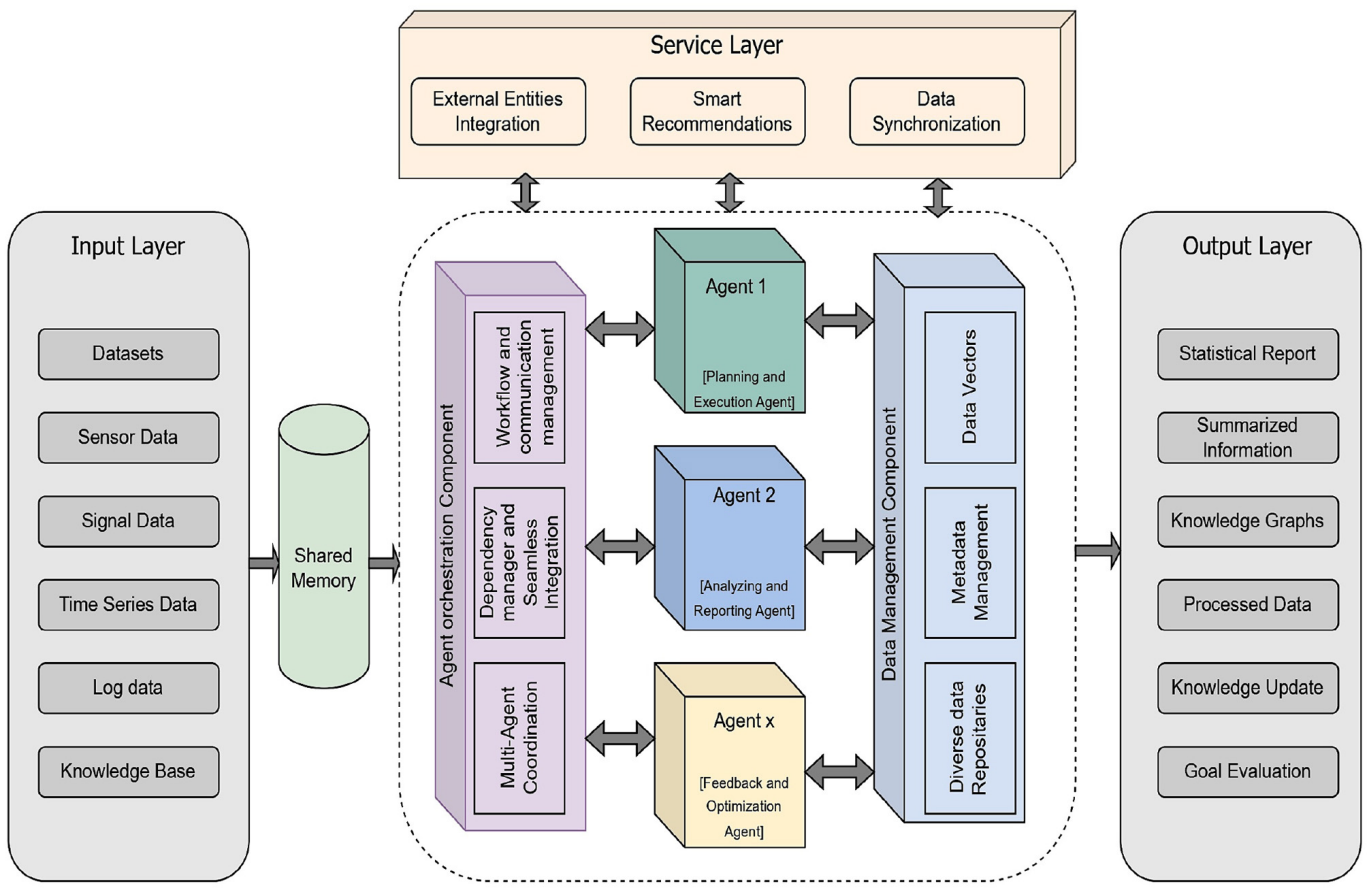
Generic architecture of the Agentic AI model. Created with Diagrams.net.

While the perception, cognitive reasoning, and action execution modules constitute the foundational architecture of an Agentic AI system, a defining characteristic lies in its capacity for continual learning and self-adaptation. This adaptive capability is enabled through continuous interaction with dynamic environments, where the system incrementally acquires data, evaluates outcomes, and refines its internal models and policies using techniques such as meta-learning (18). The architecture diagram of the Agentic AI models is being presented in Figure 2.

The architecture presented in Figure 2 is a conceptual synthesis grounded in established research across the multi-agent systems, autonomous reasoning pipelines, and cognitive AI models. The perception–reasoning–action–learning structure follows classical intelligent agent architectures (19) and is expanded upon in meta-learning and continual learning studies (20). The orchestration layer draws inspiration from multi-agent coordination and controller frameworks (21) and recent LLM-driven agentic system orchestration models. The data management and shared memory components are derived from distributed AI and blackboard system architectures widely referenced in autonomy research. Collectively, these sources inform the hierarchical and modular representation, where perception, orchestration, interaction, and learning components operate cohesively to support agentic decision-making. Therefore, the proposed architecture converges from multiple established frameworks in the literature and reflects an integrated design rather than an arbitrary structure.

The input layer of the Agentic AI model is designed to ingest heterogeneous data originating from diverse sources, including standardized datasets, sensor outputs, signal streams, time-series data, system logs, and structured knowledge bases. These multimodal inputs—characterized by their varying formats, temporal characteristics, and semantic representations (22)—are unified through an integrated shared memory architecture, enabling coherent downstream processing and cross-modal learning. The main layer consists of multiple agents, agent orchestration component, and data management component. The agent orchestration layer coordinates and manages interactions among agents to ensure efficient task execution, which is a very crucial component of the Agentic AI architecture. It is the central intelligence that manages, coordinates, and optimizes the interactions and workflows of individual agents, transforming a collection of independent AI components into a cohesive and powerful system. By effectively managing the interactions and collaboration of specialized agents, it enables the system to tackle complex, multi-phase problems that would be beyond the capabilities of a single AI model. It provides the necessary structure, control, and intelligence to ensure that the symphony of AI agents plays in harmony to achieve desired outcomes. Its responsibilities include facilitating communication, managing data flow, handling conflicts, and ensuring the overall efficient and reliable execution of multi-agent workflows to achieve system goals.

Agentic AI systems are designed to solve complex, multi-faceted problems that often require a wide range of capabilities, contexts, and domain knowledge. These systems use many specialized agents, each with a distinct role and responsibilities, to deal with these kinds of problems. These individual agents work together to get the job done better than one giant system that does everything. Each agent performs specialized functions that collectively improve system efficiency. The data management part is the most essential component of Agentic AI systems. It makes sure that all agents can handle information in a seamless, reliable, and context-aware manner. It is in charge of organizing, changing, and sharing different sorts of data that agents use to perceive, reason, and act. This component has a number of specialized subcomponents, such as data vectors, metadata management, and diverse data repositories. It provides the agents with pre-processed and relevant data on request to keep data consistent and in synchronization in shared memory architectures.

In the architecture of Agentic AI model, the service layer acts as a crucial interface, enabling agents to interact with the external environment, access diverse data sources, and leverage external capabilities. This layer is essential for agents to operate effectively beyond their internal processing and reasoning, allowing them to perceive and act upon the real world or other digital systems. Key aspects often associated with the service layer include external entity integration, data synchronization, and the deployment of capabilities such as smart recommendation systems. External entity integration connects the agent to diverse outside systems and data sources. Data synchronization ensures that the agent's internal understanding remains current by keeping its data consistent with external changes. Smart recommendation systems are an example capability that leverages this layer's access to external data for providing dynamic and context-aware suggestions.

The output layer, also called the action layer, looks after the agent's internal decisions and plans into real-time actions that happen in its environment. This layer has the equipment needed to carry out these tasks, which could be anything from using APIs to connect to digital systems to manipulating robotic effectors in the real world to creating output that people can interpret. This layer additionally has modules for post-processing to make sure that the output is precise, easy to understand, and in line with the system's goals.

## 3 Working of Agentic AI

The current section outlines the Agent Coordination and Ranking Framework (ACRF) in Agentic AI models. ACRF governs how multiple autonomous agents coordinate together to solve complex tasks, resolve dependencies, and are ranked based on performance and behavior. Furthermore, ACRF incorporates the ranking and evaluation mechanism that would continuously monitor each agent's performance using various metrics such as trustworthiness, confidence level, responsiveness, and task success rate. This performance feedback is then used to adaptively prioritize, promote, or demote agents within the system. The Escalation Unit handles scenarios where agents produce low-confidence or conflicting outputs by rerouting the task to fallback agents for resolution. The Audit Unit ensures transparency and accountability by logging agent decisions, actions, and outcomes. The components of the ACRF are presented in the Table 3.

**Table 3:** Components of agent coordination and ranking framework.

**Component**	**Description**
Agent registry	Catalog of all agents, their roles, capabilities, dependencies, and metadata
Task graph	A directed acyclic graph (DAG) representing how tasks flow through agents
Controller	Orchestrator that activates agents, passes messages, and monitors outputs
Ranking engine	Continuously scores and ranks agents based on trust, accuracy, and behavior
Audit layer	Tracks agent decisions for fairness, compliance, and traceability

Agent coordination refers to the structured mechanism by which multiple autonomous agents can interact to perform a task by coordination. Each agent is designed to handle a specific task and communicates with others via a controller. Each agent executes in the order of dependency as per the task graph. Resultantly, the output of one agent becomes the input for the next, and the controller resolves conflicts that occur during the agent selection and sequence of execution. The corresponding algorithm is presented in Algorithm 1.

Algorithm 1 Agent coordination and communication Workflow. 
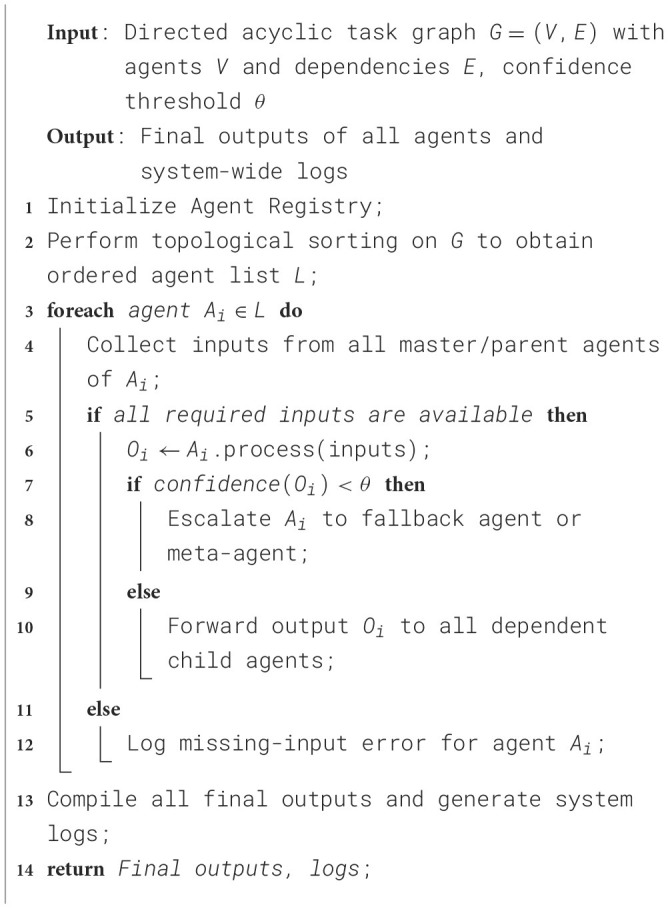

The Controller is the central orchestrator of the Agentic AI model that manages the agent lifecycle, message routing, execution flow, and ensures task correctness in an agentic system. The other key responsibilities include agent activation, monitoring outputs, fallback escalation, and error handling. The corresponding algorithm for agent controller and task manager is presented in Algorithm 2.

Algorithm 2 Agent controller and task manager. 
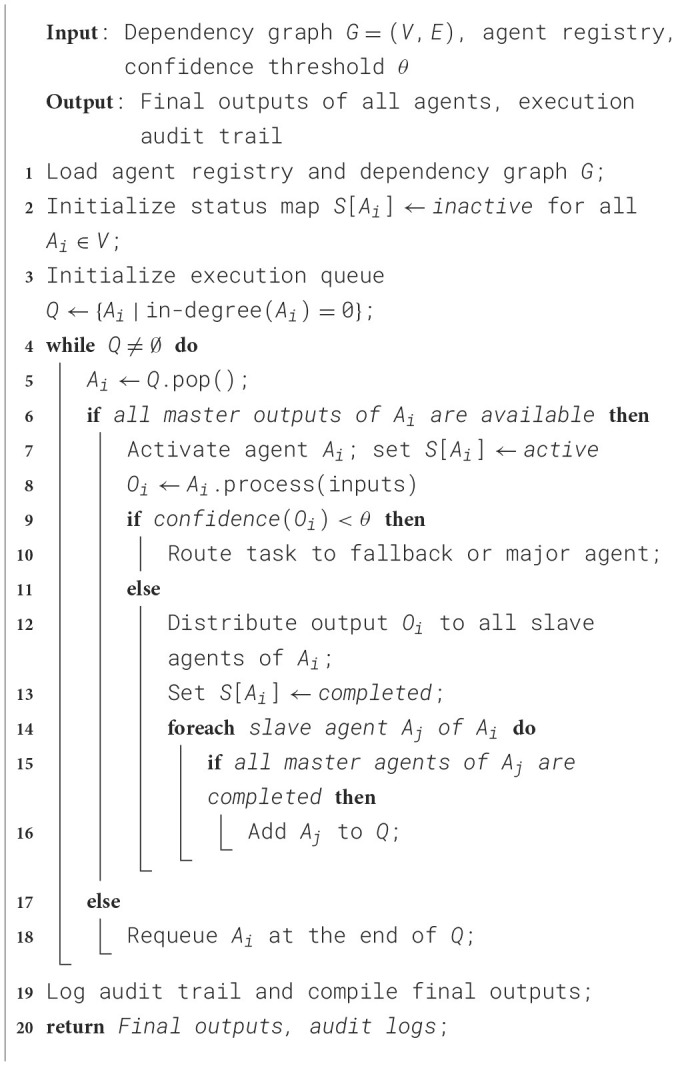

**Figure 3:**
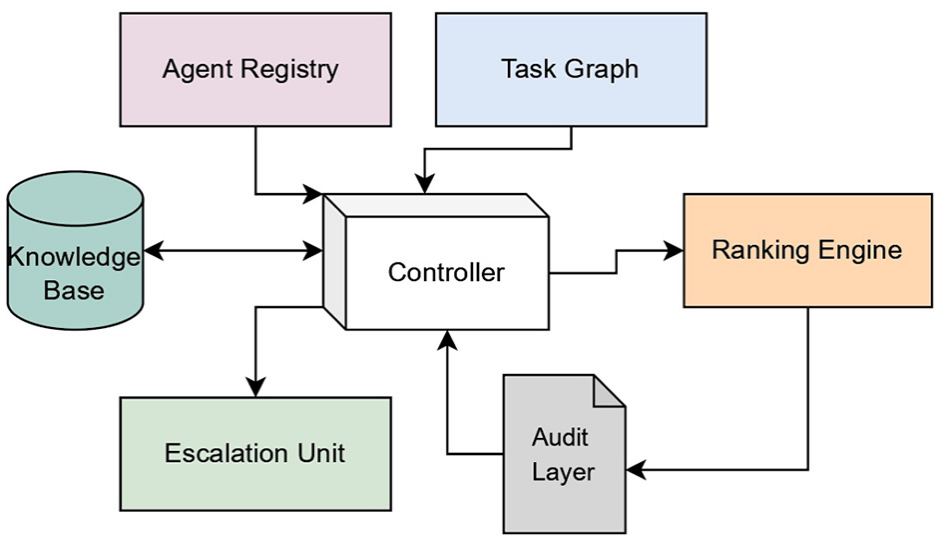
Various significant components of Agentic AI. Created with Diagrams.net.

The Ranking Engine is responsible for maintaining a dynamic trust and performance profile of each agent in the system. It would assist in guiding the agent selection process when multiple agents are available, continuously learn from feedback, and adapt agent priorities. The corresponding algorithm for the rank updating is presented in Algorithm 3. The crucial components of the Agentic AI model are presented in Figure 3.

Algorithm 3 Agent ranking and score update. 
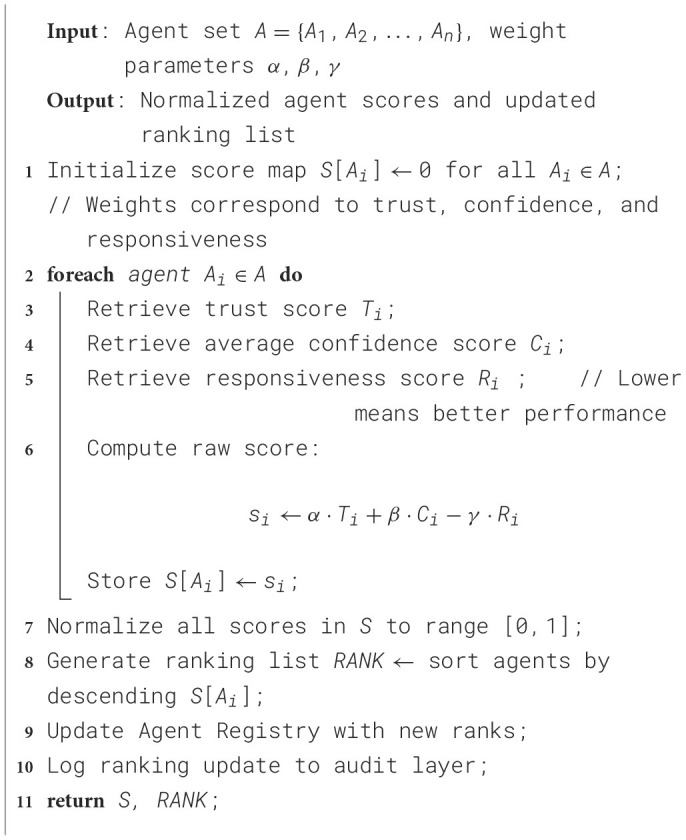

## 4 Role of Agentic AI in healthcare

Agentic AI has the potential to transform the healthcare domain by enabling intelligent and autonomous systems that can manage complex clinical workflows, including the necessary support in the decision-making process, and personalize patient care. By employing multiple specialized agents, it can disseminate tasks such as medical data analysis, patient monitoring, diagnostic support, and treatment recommendation across multiple agents. Agentic AI can operate across diverse data sources, including electronic health records (EHRs) (23), sensor data, and imaging systems. These agents collaborate to detect anomalies, predict disease progression, and optimize care plans in real-time, reducing the cognitive load on healthcare professionals. Furthermore, Agentic AI can power virtual health assistants for patient monitoring and engagement, accelerate drug discovery by analyzing complex biological data, and enhance the efficiency of hospital resource management, ultimately contributing to improved patient outcomes and a more efficient healthcare system.

### 4.1 Case study in healthcare

**Figure 4:**
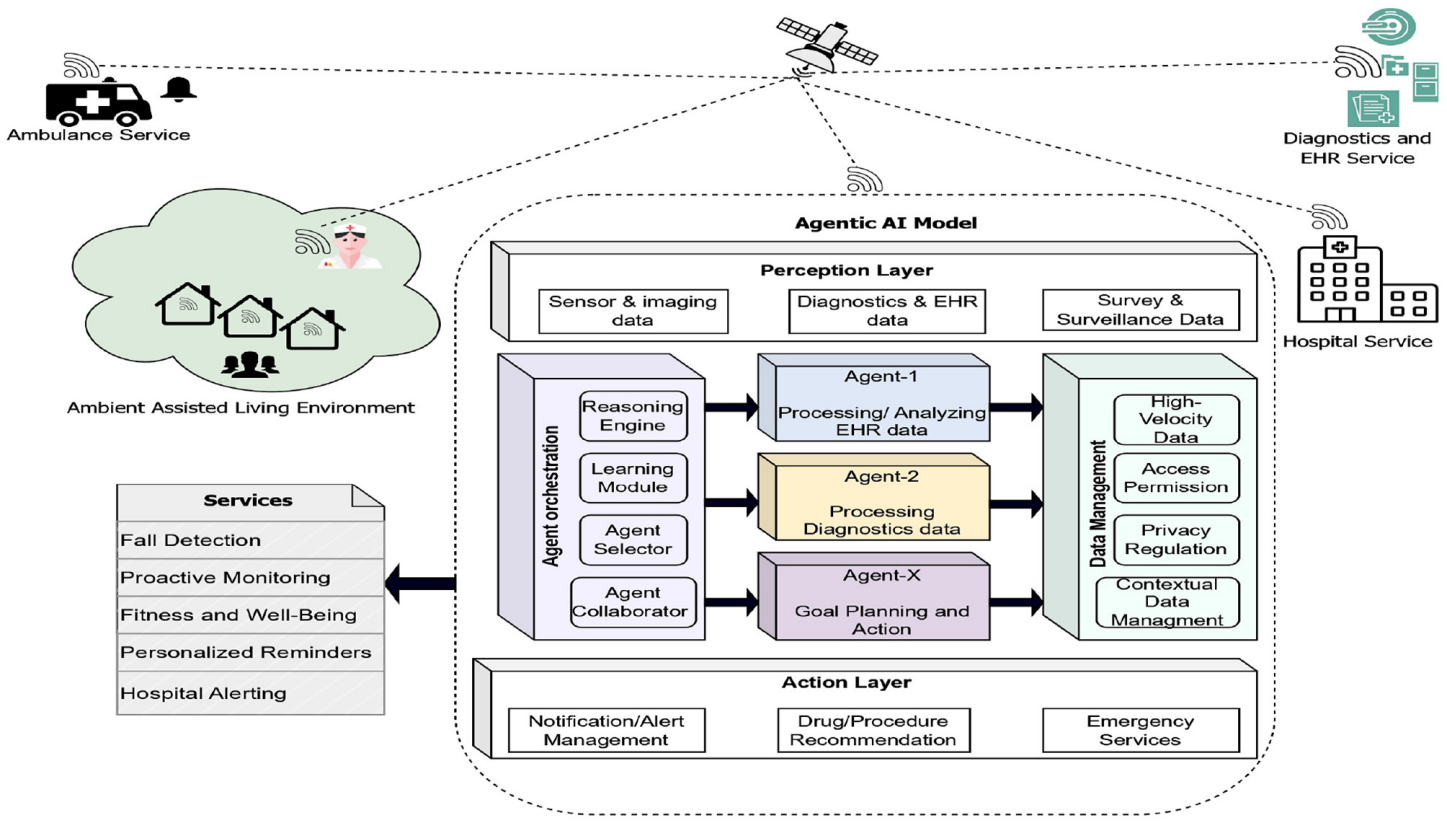
Agentic AI model in the healthcare domain. Created with Diagrams.net.

With a growing elderly population globally, there is an increasing necessity for an autonomous model that can empower older adults to live independently and safely in their homes while simultaneously addressing the burden on healthcare systems and the shortage of caregivers (24) in the Ambient Assisted Living environment (25). Agentic AI would overtake the conventional model in simultaneously handling multiple tasks, which would assist in providing precise treatment and timely care. The Agentic AI model on integrating a multi-agent architecture to provide continuous monitoring, proactive assistance, and intelligent support to the individual's needs. Agentic AI can handle the data being collected from multiple sources, such as CCTV footage data, sensor data, and signal data, being simultaneously processed. By integrating data from various sensors and systems, the Agentic AI can detect potential risks, provide timely interventions, and facilitate communication with caregivers and healthcare professionals, thereby improving the reliability and responsiveness of healthcare monitoring systems. The generic architecture of Agentic AI for the healthcare domain is presented in Figure 4.

In the above case study, the data are obtained and processed across divergent sources that include the data from diagnostics and electronic health record data, sensor and imaging data from the wearable sensors and home sensors, survey and surveillance data, hospital service data, and emergency services related data. These diverse inputs form the high-velocity and contextual data streams crucial for real-time understanding of the environment. The core components of the Agentic AI model would involve layers such as the perception layer, the agent orchestration layer, specialized agents, the data management layer, and the action layer.

The perception layer is responsible for collecting, integrating, and analyzing the data from various sources. The perception layer also performs pre-processing of the data to remove noise and inconsistencies. Fuse multiple data sources for effective monitoring of the individual to provide timely support.

The agent orchestration layer would learn from healthcare data to make intelligent decisions; it would rationalize the plan that would assist in managing the goals. It is also responsible for continuously learning individual health patterns and updating decisions and goals based on outcomes. It maintains the synchronization among multiple agents and dynamically assigns tasks to specialized agents. It manages the goals such as detecting early signs of stroke or analyzing the patterns of normal blood pressure trends in individuals. Multi-agent collaborations would look heart-monitoring agent, nutrition-monitoring agent, human activity agent to work simultaneously. Multiple agents are designated to work simultaneously across divergent domains, where one agent would analyze the EHR data, another would work on smart diagnostics, another would track human activity, and another would work on emergency care, where each of them has their own responsibilities to work collaboratively.

The data management layer is crucial in processing the data and providing the necessary input to the agent. The sensor data and real-time data need to be processed and converted into statistical data for analysis. The data need to be quantized when high-velocity real-time data ar given as input to the models. When monitoring the individual in the ambient environment, the data from the deployed sensors and CCTV footage would be of high velocity, and they obviously need a separate component to handle such data. Regulating privacy and managing access permissions is another big challenge when dealing with the sensitive data of individuals. The data management layer is responsible for managing the data privacy and the access control over the data.

The action layer is one of the other significant layers in performing the activities. This layer would perform some activities such as notification/alert management, drug/procedure recommendation, emergency services activation (26), and managing the fitness and well-being programs. This layer generates system outputs, including alerts, recommendations, and emergency actions, ensuring timely and context-aware responses. It ensures timely interventions by continuously monitoring the resident's health status and initiating preventive measures when necessary. Additionally, the action layer facilitates personalized healthcare support by delivering context-aware reminders for medication, hydration, and physical exercises. Its ability to interface seamlessly with emergency response teams further strengthens the safety net provided to vulnerable individuals.

Overall, the Agentic AI system seamlessly manages a wide spectrum of responsibilities, from supporting residents' daily activities to delivering critical healthcare interventions within an Ambient Assisted Living environment (27). Agentic AI ensures personalized, proactive, and timely support for each individual that ultimately promotes a safer, healthier, and more independent living experience.

## 5 Integration in 6G technology

**Figure 5:**
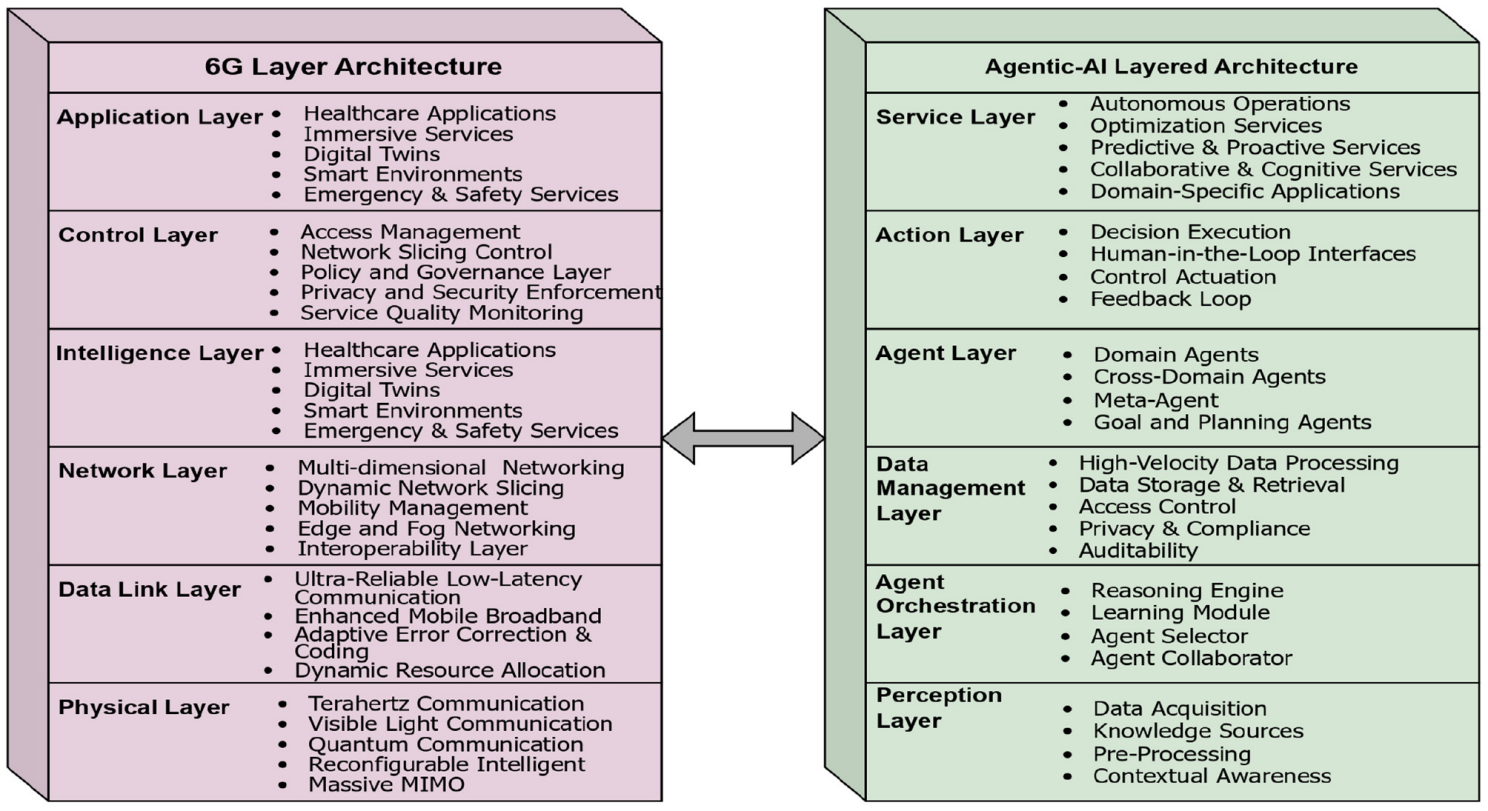
Layered architecture of 6G and Agentic AI technology. Created with Diagrams.net.

The current section would outline the role of Agentic AI over the 6G networking technology (28). The Agentic AI framework would be seamlessly deployed over the emerging 6G networking technology that will enable the ultra-reliable, intelligent, and context-aware healthcare services. By leveraging the 6G technology, native AI integration, ultra-low latency, high-speed communication, and semantic awareness are possible (29). Where the perception, orchestration, and action layers of the Agentic AI model can directly align with 6G architectural layers. For example, the Agentic AI perception layer can integrate with the physical and data layers of 6G technology, ensuring efficient acquisition and transmission of high-speed data such as sensor readings, imaging, and remote monitoring streams. The agent orchestration layer, including reasoning engines, learning modules, and collaborative agents, maps onto the 6G network intelligence and network function layer, where distributed intelligence and AI-driven decision-making are natively supported. Finally, the action layer of Agentic AI aligns with the service and application layers of 6G, enabling personalized healthcare services such as fall detection, hospital alerting, and proactive monitoring to be delivered in real-time with guaranteed quality of service (QoS) and privacy. It can be observed from the above table that the cross-layer integration allows 6G networks to act not only as communication enablers but also as cognitive collaborators in healthcare delivery (30). The layered architecture of the Agentic AI and 6G technology is presented in Figure 5.

The communication among the Agnetic AI modules and the 6G networking modules is illustrated through the Algorithm 4. More stable and smarter networking technology would have a significant impact on delivering the services in a more effective manner. Surgeons can achieve real-time control and receive high-fidelity haptic and visual feedback, essential for delicate procedures. Meanwhile, Agentic AI enhances surgical safety and precision by analyzing sensor data, predicting risks, and autonomously correcting tool trajectories when necessary. Together, they enable highly reliable, adaptive, and precise remote surgeries, expanding access to specialized healthcare across geographical boundaries, while ensuring patient safety and better clinical outcomes.

Algorithm 4 Agentic AI–6G cross-layer communication algorithm. 
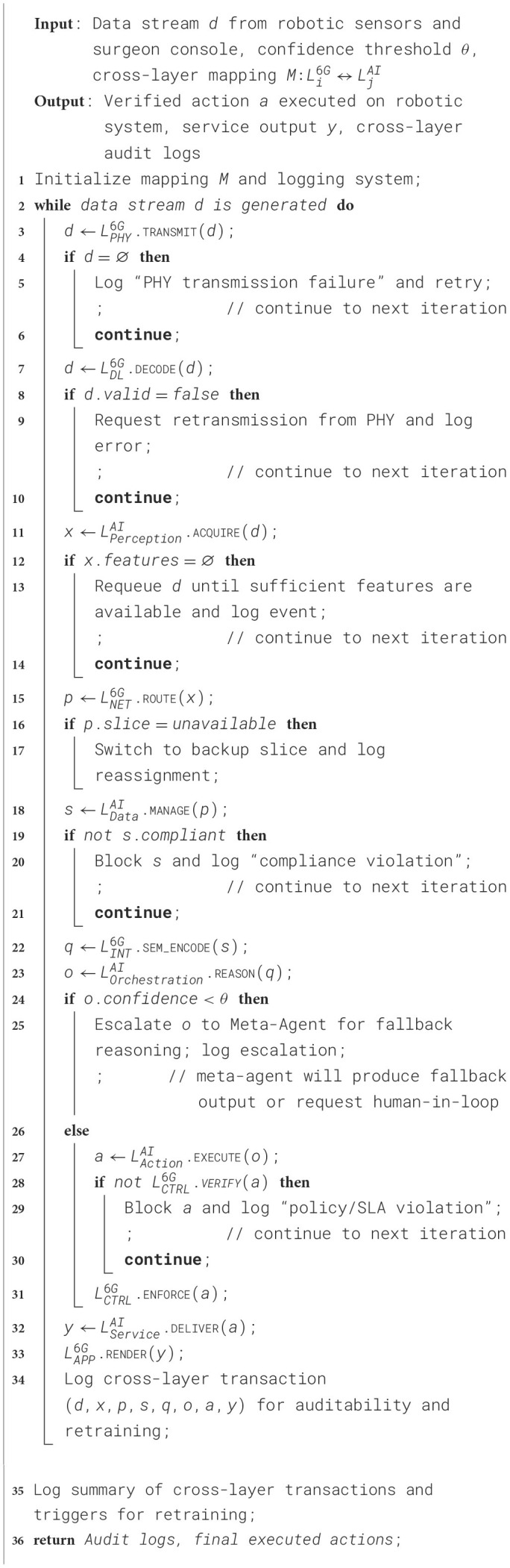

Collectively, this cross-layer synergy would significantly reduce the decision latency, improve confidence in AI reasoning, and ensure that multi-agent coordination operates in real-time even under heterogeneous and resource-constrained environments. Consequently, the integrated framework enables scalability, resilience, and energy efficiency of the system, leading to a substantial improvement in the responsiveness, accuracy, and robustness of Agentic AI-driven applications.

### 5.1 Case study in 6G

The current case study is concerned with the remote robotic surgery (31) through the integration of Agentic AI and 6G networking technologies. Remote robotic surgery has emerged as a transformative technology in modern healthcare, allowing healthcare professionals, physicians, and surgeons to collaboratively perform medical procedures on patients located thousands of kilometers away. The conventional teleoperated surgery systems face latency, bandwidth, and reliability challenges, which can compromise surgical precision and can impact patient safety. The advantages of Agnetic AI over the conventional AI-driven remote robotic surgery are presented in Table 4.

**Table 4:** Comparison between conventional AI-driven and Agentic AI-driven remote surgery.

**Feature**	**Conventional AI-driven surgery**	**Agentic AI-driven surgery**
Real-time perception of multimodal data	✓	✓
Autonomous decision-making and planning	✗	✓
Adaptive error correction during surgery	✗	✓
Surgeon-in-the-loop support	✓	✓
Continuous learning (reinforcement/federated)	✗	✓
Predictive risk assessment	✗	✓
Dependency on pre-programmed models	✓	✗
Context awareness and dynamic adaptation	✗	✓
Integration with edge + cloud via 6G	•	✓
High precision with minimal latency	✗	✓

✓ represents availability, ✗ represents non-availability, and • represents partial availability.

**Figure 6:**
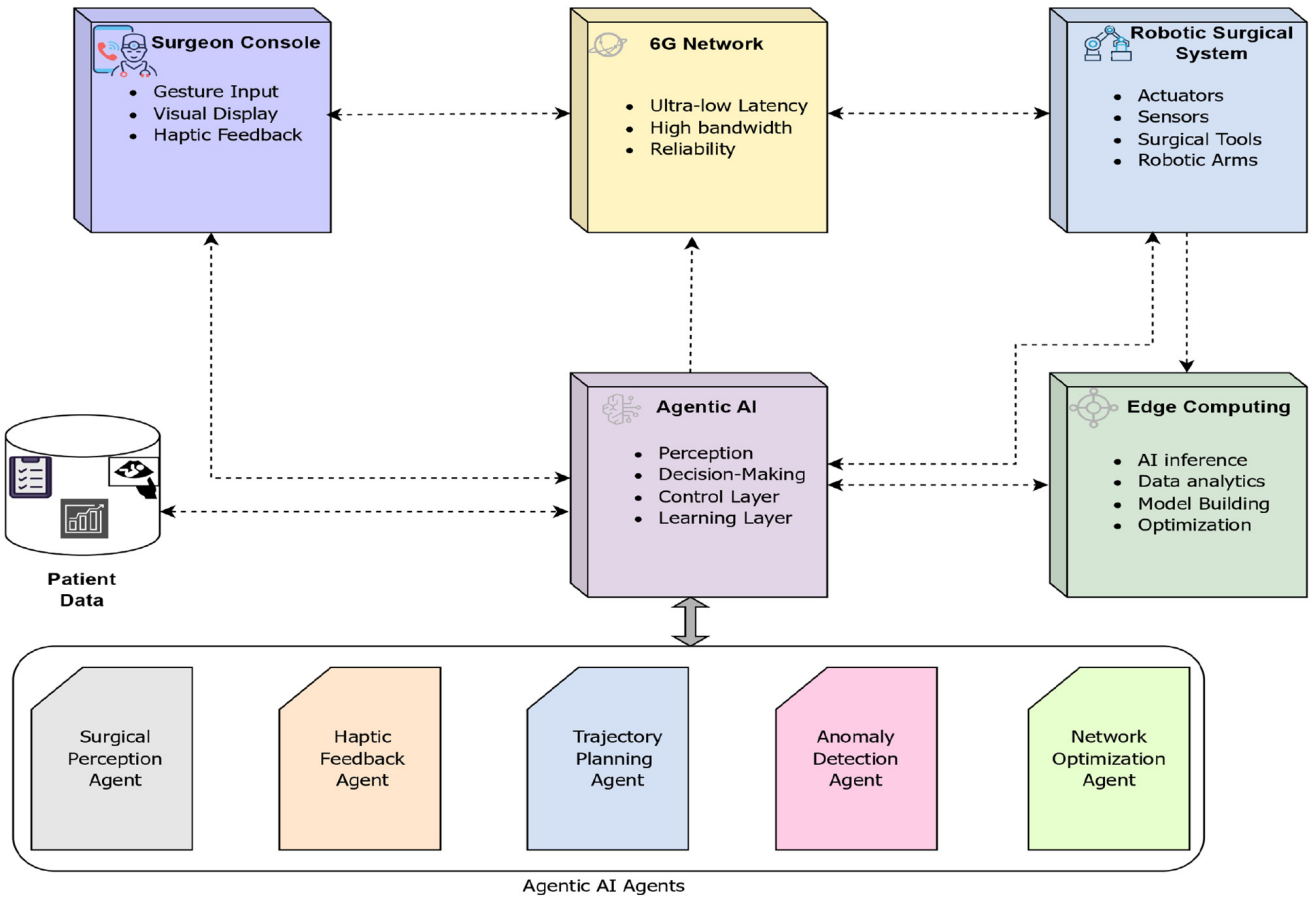
Architecture used in Remote robotic surgery over the 6G technology. Created with Diagrams.net.

With the advent of Agentic AI and integrating with 6G networks, it offers a paradigm shift by enabling autonomous surgical decision-making, real-time adaptive control, and ultra-low-latency communication. Agentic AI will refer to an autonomous agent capable of perceiving, reasoning, planning, and executing tasks with minimal human intervention. In the context of remote surgery, these agents can assist surgeons by providing predictive guidance, error correction, and adaptive tool control. Combined with 6G networking, which offers sub-millisecond latency and ultra-high reliability, the system can achieve near real-time responsiveness, critical for complex surgical procedures. The diagram representing various components of the remote robotic surgery is presented in Figure 6.

In remote robotic surgery, every Agentic AI agent takes on a focused responsibility, much like members of a surgical team working together in the operating theater. The Surgical Perception Agent interprets camera feeds and imaging scans, making sure the system appropriately identifies the tissues and vessels. Haptic Feedback Agent ensures the surgeon can feel what is happening inside the patient, even from miles away. The Trajectory Planning Agent carefully guides robotic arms to move precisely, and the Anomaly Detection Agent keeps track of patient vitals and alerts the team if something goes wrong. Supporting them, the Network Optimization Agent works behind the scenes to keep data moving smoothly over 6G. The Support Agent provides contextual guidance and decision support to the surgeon. Over time, the Learning and Adaptation Agent refines the system by absorbing knowledge from each surgery.

These agents do not work in isolation; rather, they are in constant communication, exchanging information in real-time. These agents collectively ensure operational stability, risk mitigation, and real-time decision support throughout the surgical procedure.

## 6 Agentic AI in federated learning environment

The integration of Agentic Artificial Intelligence within FL environment frameworks signifies a novel paradigm for developing decentralized, adaptable, and self-regulating intelligent systems. FL allows many distant clients, such as hospitals, IoT devices, and edge nodes, to work together to train a shared global model without having to send sensitive local data. Traditional FL frameworks, on the other hand, frequently use static coordination, limited adaptability, and centralized aggregation. This can make it hard to scale, customize, and respond to changing network conditions. Agentic AI brings in autonomous, goal-driven agents that can reason, improve themselves, and interact. This makes FL a more interactive and smarter environment.

In an Agentic FL architecture, every node that takes part operates like an intelligent agent that can learn, make decisions, and communicate with other nodes. These agents not only train models on their own data but also collaborate to achieve the best global goal, given the diverse types of data and limited resources. The agents change their strategy, including how fast they learn, how often they communicate, or how they update their models, based on how well they do locally and how the network responds. This agentic behavior makes the federated ecosystem more resilient, independent, and fair.

Multi-agent coordination algorithms that are based on swarm intelligence and collective cognition let federated systems reach decentralized consensus without having to rely on a single server. Agentic AI makes it easier to trust and understand AI by letting each agent explain its local decisions with clear reasoning trails. This is especially important in sensitive areas such as healthcare, finance, and defense. Agentic FL makes sure that both data privacy and model strength against biased or hostile behavior are protected when used with privacy-preserving technologies such as differential privacy and secure aggregation. Overall, Agentic AI gives FL systems the capacity to go from passive data-sharing frameworks to collaborative intelligence that is self-optimizing, autonomous, and in line with ethical standards. This convergence not only makes scalability and personalization better, but it also sets the stage for the next generation of networked intelligent ecosystems that can learn, negotiate, and change in a variety of settings.

**Figure 7:**
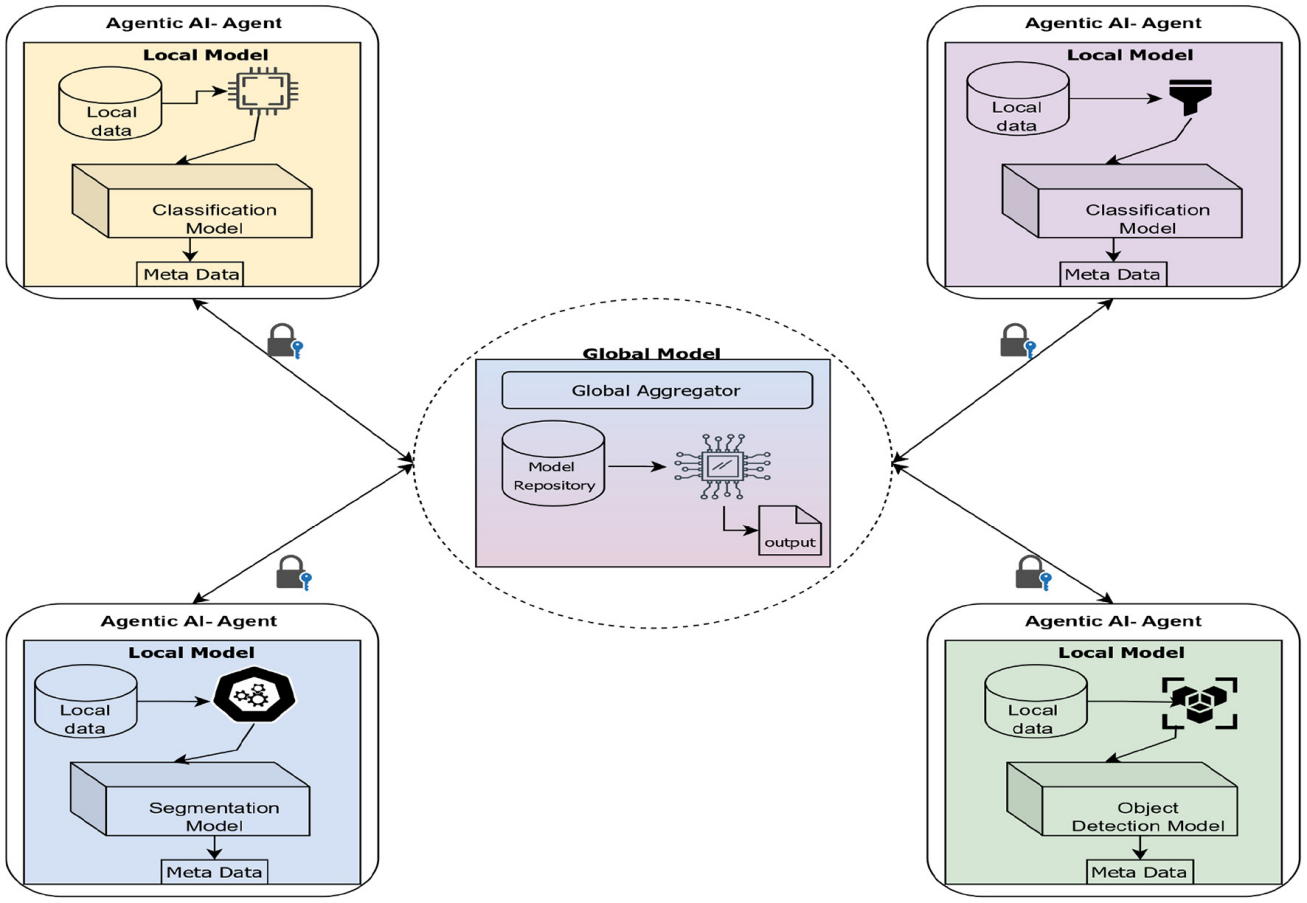
Architecture used in remote robotic surgery over the 6G technology. Created with Diagrams.net.

The architecture of Agentic AI in FL combines the autonomy and flexibility of agent-based intelligence with the privacy-preserving, distributed learning model of FL. There are three main layers: the edge agent layer, the global orchestrator layer, and the communication and security layer. The edge agent layer has smart client agents that are built into local devices or institutional servers. These agents handle data preprocessing, model training, and decision-making through modules such as the planner, learner, negotiator, communicator, and auditor. These agents work on their own on local datasets while still following global training goals. The global orchestrator layer is the backbone of coordination. It is in charge of secure aggregation, policy administration, incentive regulation, and model version control, making sure that all participants are treated fairly and that the system is stable. The communication and security layer connects these levels and makes it possible for agents to communicate encrypted models, enforce differential privacy, and check each other's trust. All of these components work together to make an ecosystem where autonomous agents work together to train a global model while keeping data sovereignty, maximizing resources, and keeping decentralized healthcare or industrial AI systems easy to understand and accountable. The architecture diagram of Agentic AI with FL is presented in Figure 7.

The global orchestrator layer is the crucial layer of the system. It controls cross-agent coordination, policy administration, and secure model aggregation. There is an Aggregation Engine that uses algorithms such as FedAvg or the FedProx, which assists in building the global models from the metadata received from the local model and controls participation and reward distribution. The communication and security layer supports necessary communication among the layers. It makes sure that encrypted model exchange happens using secure aggregation protocols, homomorphic encryption, or secure multi-party computing. This layer also handles authentication, access control, and privacy budgets that are distinct for each user to find a balance between utility and privacy. These parts work together to create a self-regulating ecosystem where autonomous agents work together to train and improve a global model without giving up data autonomy. This results in a federated intelligence that is robust and uses resources well, making it suitable for high-stakes areas such as medical imaging and smart healthcare systems. The corresponding algorithm is shown in Algorithm 5.

Algorithm 5 Communication and coordination Workflow in Agentic federated learning environment. 
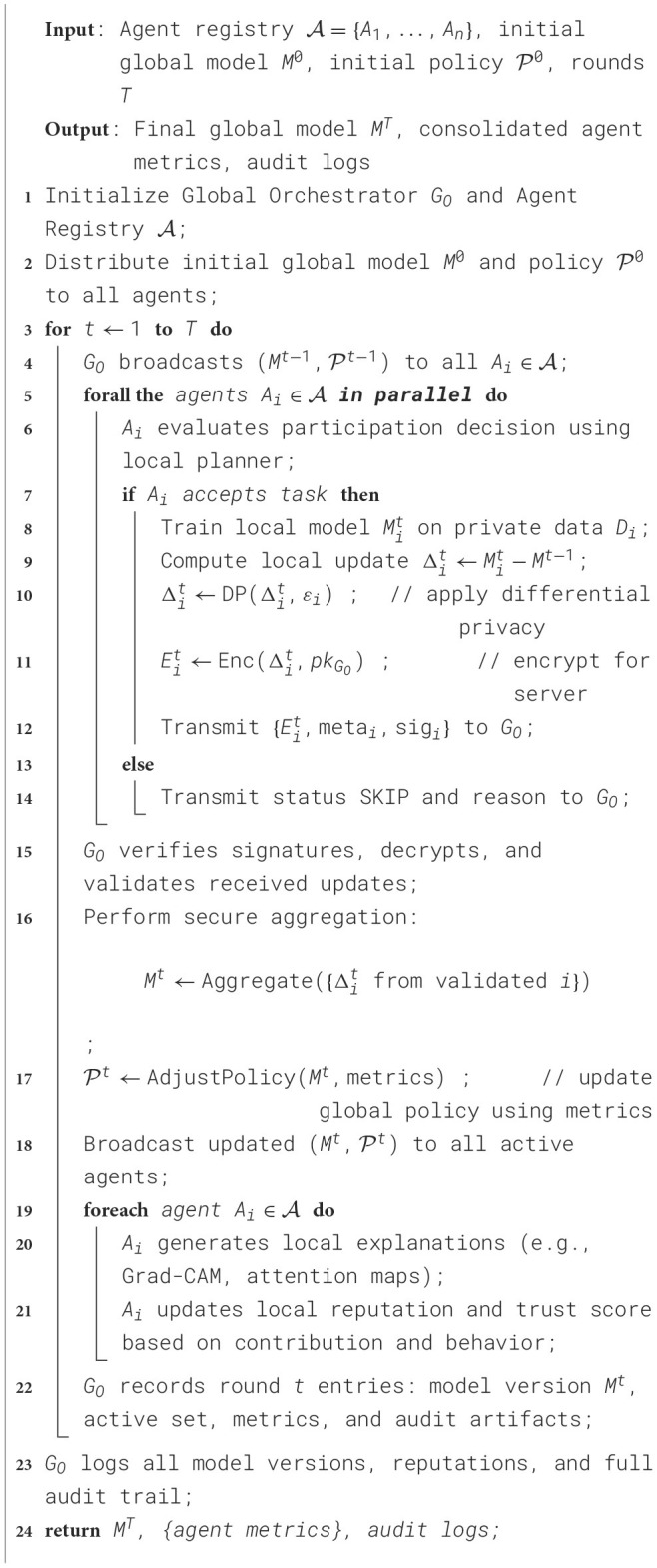

The communication and coordination algorithm creates a systematic way for autonomous agents and the global orchestrator (*G*_*O*_) to work together in a federated learning environment while maintaining privacy. The first step in each learning cycle is to share the global model and policy. Then, agents train locally at agent nodes, where data sovereignty is tightly upheld. Agents use encryption and differential privacy to send model updates to *G*_*O*_ safely. This keeps the information private and makes it harder for attackers to guess what it is. The orchestrator does validation, secure aggregation, and dynamic policy changes depending on agent trust scores and performance indicators. Feedback and explainability updates from agents strengthen transparency, accountability, and continuous enhancement. This multi-phase, agent-driven workflow allows for context-aware, adaptable, and secure learning convergence, which is the essence of Agentic AI in a federated learning model.

## 7 SWOT analysis in healthcare domain

The strengths, weaknesses, opportunities, and threats are strategic tools that would assist in the precise estimation of the capabilities of a model. SWOT analysis is performed on the suitability of Agentic AI in the healthcare domain. Each of the subsection is associated with each dimension of the SWOT analysis (32).

### 7.1 Strengths

Agentic AI has several key strengths in the healthcare domain. Its ability to proactively monitor patient health allows for the early detection of medical issues, enabling interventions before conditions worsen. Through continuous learning, the AI personalizes care by adapting to individual health patterns and daily behaviors, ensuring that each resident receives customized support. They enhance diagnostic accuracy by analyzing large, complex datasets faster and more consistently than human practitioners. These systems also improve workflow efficiency by automating routine administrative and clinical tasks, thereby reducing the burden on healthcare providers. The ability to interoperate across healthcare platforms further strengthens the healthcare ecosystem.

### 7.2 Weaknesses

Like any other supervised model, Agentic AI has some limitations. Like all other supervised learning models, the Agentic AI largely relies on the quality of the data that is fed as input for training the model (33). One of the limitation is the reliance on high-quality, annotated healthcare data, which is often difficult to obtain and may be biased. Most of the AI models are black box in nature (34), and interpreting the workings of the model is difficult, making it hard for doctors and patients to understand the rationale behind automated decisions, which can hinder trust. Additionally, the initial cost of AI system development, training, integration, and regulatory compliance is high. Moreover, the majority of the advanced technologies in AI need high-performance computers to deploy the models.

### 7.3 Opportunities

The healthcare industry presents vast opportunities for the growth of Agentic AI. There is a rising demand for predictive healthcare systems that can detect diseases early, personalize treatment, and optimize resource management at hospitals. Expanding access to wearable health technologies, genomics, and real-time patient monitoring systems offers a better personalized healthcare, which could be effective with the use of Agentic AI. The growing acceptance of telehealth and remote patient monitoring, which is accelerated by public and private sector agencies, opens up even broader usage for autonomous, agent-driven healthcare solutions.

### 7.4 Threats

Like any other AI model, the Agentic AI also has several external risks and constraints. Ethical dilemmas around patient consent, autonomy, and AI accountability continue to raise concerns among policymakers and the public. The security of the sensitive data and cyber attacks are among the threats that limit the ability of the model. Higher initial cost, adaptability, and ease of access are some of the other crucial things that are the major threats to the Agentic AI technology.

Furthermore, the summary of strengths, weaknesses, opportunities, and threats is being summarized in Table 5, providing strategic insights that can guide future implementations of Agentic AI.

**Table 5:** Concise summary of SWOT analysis for Agentic AI in healthcare.

**Strengths**	• Autonomous clinical decision support for early risk detection. • Personalized and adaptive care through continual learning. • Improved operational efficiency via workflow automation. • Scalability for multimodal, data-intensive healthcare environments. • Interoperability across IoT, cloud, and federated systems.
**Weaknesses**	• Dependence on high-quality annotated datasets. • High computational and infrastructure requirements. • Limited interpretability of agentic reasoning mechanisms. • Integration barriers with legacy healthcare systems.
**Opportunities**	• Federated and privacy-preserving learning ecosystems. • Advancements in precision medicine and patient-specific modeling. • Growth of remote, autonomous, and 6G-enabled care delivery. • Increasing industry and policy support for AI-driven healthcare.
**Threats**	• Expanded cybersecurity vulnerability in multi-agent systems. • Ethical and legal complexities in autonomous decision-making. • Risk of bias propagation and inequities in clinical outcomes. • Evolving regulatory frameworks and compliance uncertainty.

## 8 Conclusion

The present examination outlines the role of Agentic AI in the healthcare domain and clarifies its conceptual scope, aligning the work with a perspective-style contribution. The case study demonstrates how agentic systems can support a range of healthcare activities, from routine monitoring to time-critical interventions. The integration of Agentic AI with emerging communication technologies such as 6G further illustrates its potential to enhance real-time coordination and clinical decision support. While high-quality data and computational resources are important considerations for future implementation, these requirements do not limit the scope of Agentic AI to improve patient care and operational efficiency. Federated learning (FL) also contributes to this vision by enabling secure, privacy-preserving model development across distributed healthcare environments, thereby strengthening robustness and generalizability. The present study includes a SWOT analysis that highlights the strengths, challenges, opportunities, and threats associated with Agentic AI, providing a structured overview that may guide future research and system development in the healthcare domain.

## Data Availability

The original contributions presented in the study are included in the article/supplementary material, further inquiries can be directed to the corresponding author.
